# Association of Neural Responses to Drug Cues With Subsequent Relapse to Stimulant Use

**DOI:** 10.1001/jamanetworkopen.2018.6466

**Published:** 2018-12-28

**Authors:** Kelly H. MacNiven, Emily L. S. Jensen, Nicholas Borg, Claudia B. Padula, Keith Humphreys, Brian Knutson

**Affiliations:** 1 Department of Psychology, Stanford University, Stanford, California; 2 Veterans Affairs Palo Alto Health Care System, Palo Alto, Stanford, California; 3 Department of Psychiatry and Behavioral Sciences, Stanford University, Stanford, California

## Abstract

**Importance:**

Although chronic relapse is a characteristic of addiction to stimulants, conventional measures (eg, clinical, demographic, and self-report) do not robustly identify which individuals are most vulnerable to relapse.

**Objectives:**

To test whether drug cues are associated with increased mesolimbic neural activity in patients undergoing treatment for stimulant use disorder and whether this activity is associated with risk for subsequent relapse.

**Design, Setting, and Participants:**

This prospective cohort study of 76 participants included a control group for baseline group comparisons. Veteran patients (n = 36) with stimulant use disorders were recruited from a 28-day residential treatment program at the Veterans Affairs Palo Alto Health Care System. Healthy controls (n = 40) were recruited from the surrounding community. Baseline data were collected between September 21, 2015, and January 26, 2018, from patients and healthy controls using functional magnetic resonance imaging during a performance of a reward cue task. Patients’ stimulant use was subsequently assessed after treatment discharge (at approximately 1, 3, and 6 months) to assess relapse outcomes.

**Main Outcomes and Measures:**

Primary measures included neural responses to drug and food cues in estimated mesolimbic volumes of interest, including the medial prefrontal cortex, nucleus accumbens (NAcc), and ventral tegmental area. The primary outcome variable was relapse (defined as any stimulant use), assessed both dichotomously (3 months after discharge) and continuously (days to relapse). Brain activity measures were contrasted between groups to validate neural measures of drug cue reactivity, which were then used to estimate relapse outcomes of patients.

**Results:**

Relative to controls (n = 40; 16 women and 24 men; mean [SD] age, 32.0 [11.6] years), patients (n = 36; 2 women and 34 men; mean [SD] age, 43.4 [13.3] years) showed increased mesolimbic activity in response to drug cues (medial prefrontal cortex, *t*_74_ = 2.90, *P* = .005, Cohen *d* = 0.66; NAcc, *t*_74_ = 2.39, *P* = .02, Cohen *d* = 0.54; and ventral tegmental area, *t*_74_ = 4.04, *P* < .001, Cohen *d* = 0.92). In patients, increased drug cue response in the NAcc (but not other volumes of interest) was associated with time to relapse months later (Cox proportional hazards regression hazard ratio, 2.30; 95% CI, 1.40-3.79). After controlling for age, NAcc response to drug cues classified relapsers (12 patients; 1 woman and 11 men; mean [SD] age, 49.3 [14.1] years) and abstainers (21 patients; 1 woman and 20 men; mean [SD] age, 39.3 [12.3] years) at 3 months with 75.8% classification accuracy. Model comparison further indicated that NAcc responses to drug cues were associated with relapse above and beyond estimations of relapse according to conventional measures.

**Conclusions and Relevance:**

Responses in the NAcc to stimulant cues appear to be associated with relapse in humans. Identification of neural markers may eventually help target interventions to the most vulnerable individuals.

## Key Points

**Question:**

Are neural responses to drug cues associated with relapse in patients with a stimulant use disorder?

**Findings:**

In this cohort study of 76 participants, patients showed greater mesolimbic activity to drug cues than did controls, but nucleus accumbens responses to drug cues were specifically associated with the later occurrence and timing of relapse in patients to a greater extent than were conventional self-report and clinical measures.

**Meaning:**

Neural responses to stimulant cues may help identify patients at greatest risk for relapse, which could improve targeting of interventions.

## Teaser

This cohort study examines whether drug cues are associated with increased mesolimbic neural activity in patients undergoing treatment for stimulant use disorder and whether this activity is associated with risk for relapse.

## Introduction

Harmful drug use accounts for 12.4% of global mortality and 8.9% of global disability-adjusted life years,^1^ much of which can be attributed to chronic relapse. Individuals with stimulant use disorders (including cocaine use disorder and amphetamine use disorder) have a particularly severe rate of relapse: more than half of patients relapse 1 year after leaving treatment and an additional one-fourth of patients relapse 2 to 5 years after leaving treatment.^2^ Although both social (eg, unemployment) and clinical (eg, co-occurring psychiatric disorders) factors appear to increase the risk of relapse across different types of drug addiction, they do so only weakly and variably in individuals with stimulant use disorder.^3^ By discovering more robust factors associated with relapse, clinicians might better identify intervention targets and direct interventions toward the most vulnerable patients.^4^

Cues previously associated with drug use may exacerbate the risk of relapse.^5^ Theorists have suggested a number of ways that drug-associated cues could promote relapse by influencing motivational processes.^6,7^ A hijacking metaphor of drug cue action implies that long-term use of stimulants can divert motivational mechanisms from the pursuit of natural rewards toward the pursuit of drug rewards.^8^ This hijacking metaphor includes multiple channels, including sensitization, which should increase appetitive responses to drug cues,^9,10^ blunting, which should decrease appetitive responses to competing natural reward cues,^11^ and disinhibition, which should reduce control over these responses.^12^

At a neural level, animal models of cue-induced drug-seeking suggest partial mediation by mesolimbic release of dopamine.^13,14,15^ Despite the popularity of the hijacking hypothesis, however, little evidence indicates that drug cues increase mesolimbic activity in human users of stimulants.^4,16^ An early study combining positron emission tomography with a cue reactivity paradigm suggested that drug cues (videos) increased the release of dopamine in the dorsal rather than ventral striatum of individuals who used cocaine.^17^ Results from cue reactivity studies using functional magnetic resonance imaging (fMRI) in individuals who use stimulants suggest that drug cues elicit correlated activity in a number of sensory and frontal cortical regions,^18,19,20,21,22^ but no study, to our knowledge, has explicitly implicated mesolimbic activity or subsequently linked that activity to relapse. More important, these earlier studies used block designs,^20^ which may not be able to resolve second-to-second changes in neural activity associated with phasic release of dopamine.^13^ We aimed to address this gap in the literature by using an event-related design to directly test whether drug cues elicit mesolimbic responses, and whether these responses are associated with relapse in human users of stimulants.

We sought to test whether mesolimbic responses to drug cues, conventional reward cues, or both would be associated with subsequent relapse with stimulant use. To do so, we combined an event-related cue reactivity task with fMRI to visualize neural responses of patients with a stimulant use disorder and healthy control participants. We first examined whether neural responses to drug and food reward cues differed between patients and controls, and then tested whether those responses were associated with relapse in patients. To probe clinical utility, we compared associations supported by neural responses with those from more traditional individual difference and clinical measures. We hypothesized that: (1) compared with controls, patients with a stimulant use disorder would show increased mesolimbic responses to drug cues; (2) compared with controls, patients would show decreased mesolimbic responses to food reward cues; and (3) patients’ mesolimbic responses to drug and/or food cues might be associated with relapse above and beyond associations indicated by traditional measures.

## Methods

The study protocol was reviewed and approved by the institutional review boards of the Stanford University School of Medicine and Research and Development Office of the Veterans Affairs Palo Alto Health Care System. Participants provided written informed consent before participating in the study. This study followed the Standards for Reporting of Diagnostic Accuracy (STARD) reporting guidelines.

### Participants

#### Patients

Patients were recruited from a substance use disorder treatment program at the Veterans Affairs Palo Alto Health Care System, Palo Alto, California. This 28-day residential treatment program provides a substance-free living environment, access to medical care, daily group sessions (which include coping, problem-solving, motivational, and relapse prevention skills), holistic therapy options, and voluntary 12-step mutual help group meetings (eg, Alcoholics Anonymous). Urine toxicology tests were conducted throughout treatment to verify abstinence. On admission to the program, all patients underwent a full history and physical interview by clinicians (including a psychiatrist, psychiatry residents, clinical psychologist, or social workers), which included diagnostic queries about past and current psychiatric and/or substance use disorder based on *Diagnostic and Statistical Manual of Mental Disorders* (Fifth Edition) (*DSM-5*) criteria. Researchers then recruited interested and eligible patients with current substance use disorder diagnoses for stimulant drugs (eg, methamphetamine and crack and powder cocaine). Although some patients were mandated by a court to receive treatment or were conditionally released from jail into treatment, the screening tool used to determine eligibility confirmed that problems with stimulant use were the primary reason for seeking treatment. The final sample included 28 methamphetamine users and 14 crack or powder cocaine users (6 patients met the criteria for abusing more than 1 stimulant). Patients were excluded if they took medications that influence vasoreactivity and/or cerebral perfusion (eg, cardiac medications) or central dopaminergic activity (eg, stimulants or antipsychotics), had a history of traumatic head injury, if they had a history of mania or psychosis, or reported safety contraindications to undergoing standard magnetic resonance imaging (MRI) (eg, magnetic material in the head).

Patients were enrolled in the treatment program for a mean (SD) of 17.9 (1.4) days and reported most recently using a stimulant a mean (SD) of 61.4 (10.4) days (range, 8-239 days) prior to undergoing MRI as part of the study. Three of the final sample of 36 patients required medical detoxification in an inpatient psychiatric unit prior to admission into the treatment program. Most patients reported additional substance use (other than stimulants) prior to treatment (29 of 36 [81%]), and more than half of the patients had a comorbid alcohol use disorder (19 of 36 [53%]). Urine toxicology and breathalyzer tests were administered to patients immediately before undergoing MRI to detect recent use of stimulants (cocaine or amphetamines), opiates, benzodiazepines, tetrahydrocannabinol, and/or alcohol, and 3 recruited patients did not pass this final screening, leading to exclusion. All patients included in the final sample had negative test results for these substances.

#### Controls

Healthy control participants were recruited from Stanford University’s Paid Psychology Experiments pool and the surrounding community. The same exclusion criteria were applied to the control group as described above for patients, with the additional requirement that controls reported no current or past substance use disorder. A subset of these controls were US military veterans (n = 12). See eFigure 1 in the Supplement for the participant flow diagram.

For all participants, self-reported demographic variables (eg, age, sex, race/ethnicity, and educational level) were assessed to confirm comparability across groups (eTables 1 and 2 in the Supplement). Consecutive sampling was used to enroll eligible participants in both groups.

### Setting

Baseline neuroimaging data were collected between September 21, 2015, and January 26, 2018, at the Stanford Center for Cognitive and Neurobiological Imaging, Stanford, California. Patient follow-up interviews were conducted in person at the Stanford Psychology Department and via telephone from November 30, 2015, through March 27, 2018.

### Procedures

**Figure 1.**
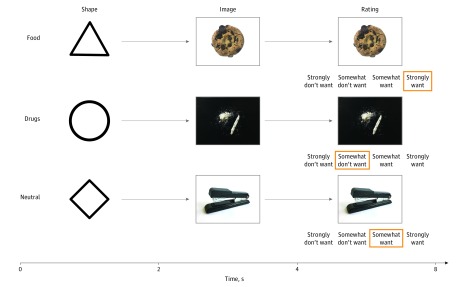
Cue Task Trial Structure Each row depicts sequential phases in trials of different conditions. Each trial began with the presentation of an abstract shape (2 seconds), followed by the presentation of an associated image (6 seconds). Two seconds after image onset, wanting ratings appeared below the image (on a 4-point Likert scale, where 1 indicates strongly do not want and 4 indicates strongly want, counterbalanced right to left), and participants rated how much they wanted the depicted item. A fixation cross then indicated a variable intertrial interval (2, 4, or 6 seconds) lasting until the beginning of the next trial.

We designed a novel cue reactivity task in which participants viewed abstract shapes that preceded images of stimulant drugs, alcohol, food, or everyday objects (Figure 1 and eAppendix 1 and eFigure 2 in the Supplement). Images of alcohol were also presented for a separate study on alcohol use disorder and thus were not included in analyses presented in this article. Shapes reliably preceded images to elicit anticipatory effect and associated brain activity before the categorically specific associated images were revealed, as in previous research.^23^ The task consisted of 18 trials of each cue type. After undergoing MRI, participants rated each image on 7-point scales indexing valence (where 1 indicated very negative and 7 indicated very positive), arousal (where 1 indicated very low and 7 indicated very high), and familiarity (where 1 indicated not at all familiar and 7 indicated very familiar). Valence and arousal ratings were later transformed to positive and negative arousal ratings.^24^ Patients additionally completed the Brief Addiction Monitor questionnaire, which assesses psychological factors (eg, craving and negative affect) as well as behaviors relevant to substance dependence during the past month.^25^

Patients’ use of stimulants was assessed approximately 1, 3, and 6 months after completing treatment using the Timeline Followback method^26^ (which shows moderate to high consistency with results of urine toxicology screening^27^; see eAppendix 1 in the Supplement for follow-up procedure details). Because treatment was abstinence based, relapse was defined as any stimulant use in the time since treatment. We chose this definition of relapse as our dependent measure rather than more broadly defining relapse as use of any drug of abuse because we were specifically interested in testing whether brain responses to stimulant drug cues were associated with future use of this category of drugs. Two treatment outcomes were considered: relapse as a binary measure (ie, a yes or no response at the 3-month follow-up) and time to relapse (ie, continuous time in days until a yes response). Additional steps were taken to minimize the clinical risks of performing a cue reactivity study (eAppendix 1 in the Supplement).

### Statistical Analysis

Preprocessing of fMRI data was conducted with a standard analytic pipeline using Analysis of Functional NeuroImages (AFNI) software^28^ (eAppendix 1 in the Supplement). Analyses included both whole-brain and volume of interest (VOI) approaches, with the latter testing critical hypotheses. For whole-brain analyses, a general linear model was fit to each voxel time series that included task-related regressors as well as nuisance regressors (eAppendix 1 in the Supplement). Trials of food vs neutral cues, trials of drug vs neutral cues, and trials of drug vs food were then contrasted, producing 3 contrast maps for each participant. Two-tailed *t* tests were performed on map coefficients to assess within-group and between-group effects, and resulting *t* maps were then *Z* transformed. Corrections for multiple comparisons were determined using a brain mask with the AFNI program 3dClustSim (AFNI, version 18.0.25; National Institute of Mental Health Scientific and Statistical Computing Core). At a voxel-based threshold of *P* < .001, clusters with 23 or more contiguous voxels (ie, 561 mm^3^) were deemed significant at *P* < .05, corrected. This corrected cluster size threshold was more stringent than the cluster size determined from nonparametric permutation tests.^29^

Volume of interest analyses tested for changes in neural activity in specific mesolimbic circuit foci implicated in previous studies of choice,^30^ which included the bilateral medial prefrontal cortex (MPFC), nucleus accumbens (NAcc), and ventral tegmental area (VTA) (eAppendix 1 in the Supplement). Time courses of normalized activity were extracted from each of these VOIs, and a mean was calculated by trial type (ie, drug, food, and neutral cues) within each participant. We then tested for differences using analysis of variance, with group (patient or control) as a between-individuals factor and cue type (food, drug, neutral cue) as a within-individuals factor, followed by targeted comparisons of differences: specifically, that patients would show increased activity to drug cues but decreased activity to food cues relative to controls.

To estimate binary incidence of relapse (defined as either present or absent 3 months after treatment), mesolimbic VOI coefficient data in patients were submitted to a series of logistic regression models along with demographic, clinical, and self-report measures. Comparison models included demographic and clinical variables, self-report variables (ie, want ratings from fMRI task and overall craving and general negative mood from the Brief Addiction Monitor questionnaire), brain activity (ie, coefficients from MPFC, NAcc, and VTA VOIs), and the strongest indices from each of these models in combination. Models were then submitted to leave-one-individual-out cross-validation to assess classification accuracy, and fits to the data (indexed by the Akaike information criterion [AIC]) were compared across models.^31^ Training sets were oversampled to contain even numbers of early abstainers and relapsers, setting the baseline probability of correctly classifying each held-out test patient to 50%. We then directly compared classification performance of the best neural risk factor (defined as the index test) associated with self-reported craving (defined as a reference standard implicated in relapse by the literature^10,32^) with an area under the curve metric. To estimate continuous length of abstinence, VOI coefficient data for NAcc responses to drug cues were submitted to survival analysis using a Cox proportional hazards regression model.

To assess whether activity in other brain regions was associated with relapse, we conducted an exploratory classification analysis on patients’ whole-brain data. Binary classifiers were trained to distinguish patients who relapsed vs abstained by applying a linear support vector machine classifier with recursive feature elimination (SVM-RFE^33^) to patients’ whole-brain coefficient data modeling neural responses to drug cues (eAppendix 1 in the Supplement).

## Results

Thirty-nine patients with a diagnosis of stimulant use disorder (ie, cocaine use disorder and/or amphetamine use disorder) and 42 healthy controls participated in the study. Data from 3 patients and 2 controls were excluded because of excessive head motion (ie, >1-mm movement from 1 whole-brain volume acquisition to the next in >1% of whole-brain volumes acquired), leaving 36 patients (2 women and 34 men; mean [SD] age, 43.4 [13.3] years) and 40 controls (16 women and 24 men; mean [SD] age, 32.0 [11.6] years) for analysis (eFigure 1 and eTables 1 and 2 in the Supplement).

### Behavior

Analysis of variance confirmed that group (patient or control; between-individuals factor) and cue type (food, drug, or neutral cue; within-individuals factor) influenced self-reported ratings collected both during and after the MRI as hypothesized (eAppendix 2 and eFigure 3 in the Supplement). Post hoc *t* tests confirmed that both controls and patients reported wanting food cues more than neutral cues (controls, *t*_39_ = 5.77; *P* < .001; Cohen *d* = 0.91; and patients, *t*_35_ = 6.55; *P* < .001; Cohen *d* = 1.09) and drug cues less than neutral cues (controls, *t*_39_ = −13.86; *P* < .001; Cohen *d* = −2.19; and patients, *t*_35_ = −2.14; *P* < .001; Cohen *d =* –0.36). Patients, however, still reported wanting drug cues more than did controls (*t*_74_ = 5.61; *P* < .001; Cohen *d* = 1.28).

### Brain Activity

**Figure 2.**
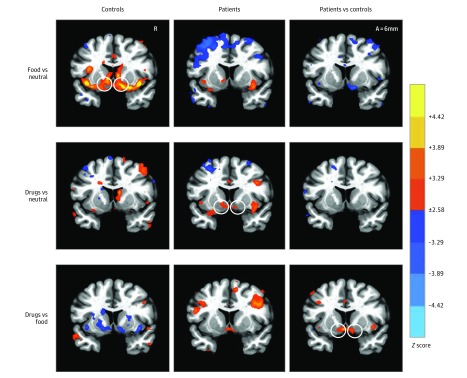
Contrasts of Neural Responses to Food, Drug, and Neutral Cue Trials in Patients and Controls Whole-brain maps (coronal view, 6 mm anterior [A] to the anterior commissure) show activations at a voxelwise threshold of *P* < .01 (uncorrected for display; each color increment depicts an order of magnitude increase). Activation maps specifically depict contrasts for controls (left), patients (middle), and patients vs controls (right). Circles highlight predicted contrasts in nucleus accumbens volumes of interest. R indicates right.

**Figure 3.**
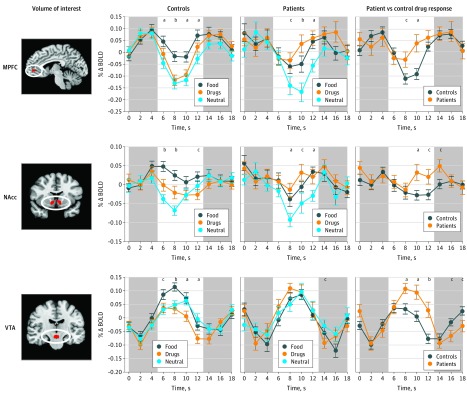
Mesolimbic Volume of Interest Responses to Different Cue Stimuli for Patients and Controls Activity time courses were extracted from predefined volumes of interest and averaged by trial type to compare responsiveness in patients vs controls. Controls showed a higher response to food stimuli than drug and neutral stimuli in all volumes of interest (middle left). Patients showed a higher response to food and drug stimuli than neutral stimuli in the medial prefrontal cortex (MPFC) and nucleus accumbens (NAcc), but not the ventral tegmental area (VTA) (middle right). Patients showed a higher response to drug cues than controls in all mesolimbic volumes of interest (right). Circles represent means and error bars depict SEM across participants. The x-axes represent time elapsed from trial onset, with white areas corresponding with the time of trial presentation (2-second shape presentation, 2-second image presentation, and 4-second rating period; shifted to account for a 6-second hemodynamic lag). In control and patient plots, *P* values indicate results of repeated-measures analysis of variance testing for differences across cue stimuli. In patient vs control drug response plots, *P* values indicate results from 2-sample *t* tests. All *P* values are 2-tailed. Δ indicates change; BOLD, blood oxygen level dependent activity. Areas in red within ovals represent volumes of interest. ^a^*P* < .01. ^b^*P* < .001. ^c^*P* < .05.

Whole-brain contrasts compared neural responses to food, drug, and neutral cues in patients and controls (Figure 2 and eFigure 4 and eTable 3 in the Supplement). The critical contrast of drug vs food cues provided the most precise test of differential group responses consistent with the hijacking hypothesis. Direct comparison of the drug vs food cue contrasts for patients vs controls confirmed increased activity in mesolimbic regions (ie, VTA and NAcc), as well as in the left middle and medial frontal gyri in patients. Analyses of activity time course data tested specific hypotheses about targeted mesolimbic VOIs (including the bilateral MPFC, NAcc, and VTA; Figure 3). All 3 VOIs (averaged across volume acquisitions occurring 6-12 seconds after trial onset) showed significant group by cue type interactions (MPFC, *F*_2,148_ = 4.3; *P* = .01; ε^2^ = 0.037; NAcc, *F*_2,148_ = 5.3; *P* = .006; ε^2^ = 0.043; and VTA, *F*_2,148_ = 7.2; *P* = .001; ε^2^ = 0.067). Post hoc *t* tests confirmed that relative to controls, patients showed increased responses to drug cues in all 3 VOIs (MPFC, *t*_74_ = 2.90; *P* = .005; Cohen *d* = 0.66; NAcc, *t*_74_ = 2.39; *P* = .02; Cohen *d* = 0.54; and VTA, *t*_74_ = 4.04; *P* < .001; Cohen *d* = 0.92), decreased responses to food cues only in the NAcc (MPFC, *t*_74_ = −1.81; *P* = .07; Cohen *d* = −0.41; NAcc, *t*_74_ = −2.67; *P* = .009; Cohen *d* = −0.61; and VTA, *t*_74_ = −1.57; *P* = .12; Cohen *d* = −0.36), and no difference in neural responses to neutral cues (MPFC, *t*_74_ = −0.58; *P* = .56; Cohen *d* = −0.13; NAcc, *t*_74_ = –0.94; *P* = .35; Cohen *d* = −0.21; and VTA, *t*_74_ = 0.84; *P* = .40; Cohen *d* = 0.19).

### Modeling Relapse

Three months after completing treatment, 12 patients had relapsed (1 woman and 11 men; mean [SD] age, 49.3 [14.1] years), while 21 remained abstinent (1 woman and 20 men; mean [SD] age, 39.3 [12.3] years). Logistic regression models tested the extent to which demographic and clinical factors, self-report measures, and brain activity were associated with relapse at 3 months (Table). Each demographic or clinical factor (which included but was not limited to diagnoses of depression, anxiety, and posttraumatic stress disorder; use history of alcohol, marijuana, and opioids; duration of stimulant use; days abstinent; and days in treatment prior to participation) was independently tested for its association with treatment outcome (eTable 2 in the Supplement). None of the factors were associated with relapse except for age (AIC, 42.95; *R*^2^ = 0.130). Although duration of use was correlated with age (*r* = 0.55; *P* = .001), only age was significantly associated with relapse.

**Table.** Regression Models of Factors Associated With Subsequent Relapse at 3 Months Among Patients With a Stimulant Use Disorder

Factor	Standardized Regression Coefficient (SE) [*Z* Score]			
	Demographic or Clinical	Self-report	Neural	Combined
Intercept	−0.63 (0.39) [−1.61]	−0.54 (0.38) [−1.43]	−0.75 (0.45) [−1.67]	−0.73 (0.45) [−1.62]
Age	0.80 (0.41) [1.96]^a^	NA	NA	0.87 (0.50) [1.74]^b^
Drug wanting	NA	−0.49 (0.47) [−1.06]	NA	NA
Craving	NA	0.41 (0.41) [1.00]	NA	NA
Negative mood^c^	NA	0.45 (0.44) [1.03]	NA	NA
MPFC drug	NA	NA	−0.64 (0.46); [−1.37]	NA
NAcc drug	NA	NA	1.45 (0.57) [2.55]^a^	1.34 (0.56) [2.38]^a^
VTA drug	NA	NA	0.44 (0.46) [0.97]	NA
Pseudo *R*^2^	0.130	0.064	0.300	0.332
AIC	42.95	48.23	39.82	36.95
Classification accuracy (leave-one-individual-out), %	66.7	48.5	69.7	75.8

Abbreviations: AIC, Akaike information criterion; MPFC, medial prefrontal cortex; NA, not applicable; NAcc, nucleus accumbens; VTA, ventral tegmental area.

^a^ *P* < .05.

^b^ *P* < .10.

^c^ Based on responses to question 3 of the Brief Addiction Monitor questionnaire.^25^

In a model including self-report measures (ie, craving, negative affect, and drug wanting), no variables were significantly associated with relapse (AIC, 48.23; *R*^2^ = 0.064). In a model that included neural variables, only NAcc response to drug cues was associated with relapse (AIC, 39.82; *R*^2^ = 0.300). Responses in the NAcc to drug cues were comparably associated with relapse at 1 and 6 months after treatment (eTable 4 in the Supplement), suggesting that this association remained stable during the assessment window. An alternative model including both NAcc responses to drug cues and food cues indicated that inclusion of responses to food cues did not significantly improve model fit (change in AIC, 1.4). Finally, a model that combined the most robust factors from separate models revealed that the NAcc response to drug cues continued to be associated with relapse above and beyond factors derived from demographic and self-report variables, and this combined model accounted for slightly more variance with a better fit than other models (AIC, 36.95; *R*^2^ = 0.332) (Table). Classification accuracy for this combined model was 75.8%. Furthermore, direct comparison of single-term models yielded an area under the curve of 77.0% for NAcc response to drug cues, compared with an area under the curve of 57.7% for self-reported craving (eFigure 5 in the Supplement).

**Figure 4.**
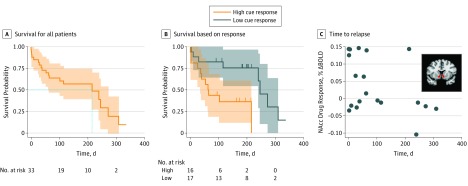
Association of Nucleus Accumbens (NAcc) Response to Drug Cues With Incidence of Relapse and Time to Relapse A, Kaplan-Meier survival curves for all patients. B, Kaplan-Meier survival curves based on high and low NAcc responses to drug cues (median split for purposes of illustration). C, Time until relapse plotted as a function of baseline NAcc volume of interest (inset) response to drug cues. BOLD indicates blood oxygen level dependent activity.

To characterize the association between NAcc responses to drug cues and time to relapse, we conducted a survival analysis with Cox proportional hazards regression, treating days to relapse as a continuous outcome. Consistent with analyses of incidence, NAcc activity elicited by drug cues was associated with an elevated risk to relapse sooner (hazard ratio, 2.30; 95% CI, 1.40-3.79; *P* = .001) (Figure 4).

To check whether activity in other brain regions was associated with relapse, whole-brain classifiers (ie, SVM-RFE with leave-one-individual-out cross-validation) identified drug response coefficient features that best classified patients who relapsed vs those who abstained (at C = 10.00, 5% of selected features with leave-one-individual-out cross-validated test accuracy of 60.0%). Back-projection of these features into a standard brain space confirmed that they included clusters of coefficients in the bilateral NAcc (eFigure 6 and eTable 5 in the Supplement). Because the classification rate of this “model-free” whole-brain analysis did not exceed that of hypothesized VOIs, however, the findings confirmed but could not supersede targeted VOI models.

To examine why neural indices could anticipate relapse in the combined model but self-report indices did not, a follow-up exploratory analysis tested for dissociations between brain activity and self-report measures in patients, potentially consistent with reduced insight.^34^ A 2 × 3 (group: patient, control [between-individuals factor]; and cue: neutral, food, drug [within-individuals factor]) analysis of variance on coefficients representing the association between NAcc responses and positive arousal ratings for each picture revealed a significant main effect of group (*F*_1,122_ = 4.9; *P* = .03; ε^2^ = 0.023), but no significant main effect of cue type (*F*_2,122_ = 0.1; *P* = .94) or interaction (*F*_2,122_ = 0.1; *P* = .90), suggesting that controls showed a stronger association of NAcc responses with positive arousal ratings than did patients for all cue types (eFigure 7 in the Supplement). Although exploratory, this finding is consistent with accounts in which patients show reduced reflective insight into their neural affective responses, rather than supporting a more targeted dissociation only between self-reported affect and neural responses to drug cues.^34^

## Discussion

Neuroimaging of a reward cue task revealed that drug cues increased activity in mesolimbic regions (including the MPFC, NAcc, and VTA) in patients recovering from a stimulant use disorder relative to controls. Patients also showed slightly decreased NAcc activity in response to conventional reward cues (ie, food) relative to controls. Longitudinally, increased NAcc responses to drug cues in patients anticipated both the incidence and speed of relapse above and beyond estimations supported by clinical, self-report, and demographic measures, suggesting that neuroimaging data can add value by providing unique and novel information about relapse. Model-free classification analyses reproduced but did not improve on analyses using targeted brain activity. Together, in patients with a stimulant use disorder, these findings suggest not only that neural activity indexes specific reactions to drug cues, but moreover, that a subset of these neural responses are associated with relapse.

This research makes several novel contributions. First, consistent with the notion of hijacking, the findings supported a sensitization mechanism of long-term use of stimulants.^10^ Previous drug cue studies of patients with stimulant use disorders have not used event-related pseudorandom designs, and so may have been unable to resolve phasic mesolimbic responses to unexpected events.^4,24^ The event-related design in our study, however, revealed that patients did show increased mesolimbic responses (ie, in the VTA, NAcc, and MFPC) to drug cues relative to controls. The localization of these enhanced drug cue responses to mesolimbic regions rather than the sensorimotor cortex implies that this sensitization may be affective rather than sensory or motor in nature. Follow-up analyses, however, hinted that affective potentiation may prove difficult to detect because of a decoupling of self-report with mesolimbic responses in patients relative to controls.^34,35^

Second, inclusion of multiple control conditions involving conventional reward cues (eg, for appetizing foods) and neutral cues provided some support for a blunting mechanism of long-term use of stimulants. Although both controls and patients showed mesolimbic responses to food cues, these responses were diminished in patients, despite similar responses to neutral cues. These findings are consistent with research indicating that patients with substance use disorders may show slightly blunted responses to conventional reward cues (eg, money),^4,36,37^ which may or may not precede experience with drugs of abuse.^31^

Third, the longitudinal design allowed us to test whether neural responses to drug cues were also associated with subsequent relapse. Of all targeted mesolimbic regions, only NAcc responses to drug cues were associated with relapse, both with respect to incidence at 3 months, and with respect to timing. Mesolimbic responses to food reward cues, however, did not improve these estimations. Furthermore, a statistical classifier trained on whole-brain responses to drug cues did not outperform a simpler regionally specific model. These initial findings parallel recent evidence that NAcc responses to alcohol cues are associated with incidence of relapse in patients treated for alcohol use disorders.^38^ Although most patients were assessed for relapse by telephone, a subset were interviewed and underwent urine drug screening in person, which yielded results that concurred with self-reported relapse status.

Fourth, quantitative comparisons of novel neural markers vs traditional clinical and behavioral assessments revealed that the neural markers could add value and might serve as a “neurophenotype” of the risk of relapse in individuals with stimulant use disorders.^39^ In the current sample, self-report measures (eg, of affect, wanting, and craving) were not significantly associated with relapse among patients. An exploratory analysis correlating positive arousal ratings with brain activity across all stimuli revealed that NAcc activity was more strongly associated with self-reported ratings in controls than in patients. These findings imply that patients may have less awareness of or access to neural markers of motivation.^34,35^ Thus, measures of brain activity might reveal clinically significant information in patients, even if they lack insight or conscious awareness into their own motivation. Neural information might therefore aid clinicians in planning and focusing treatment resources. The cost-effectiveness of using neural markers in clinical practice, however, remains to be established.^3^

Fifth, these findings help to bridge comparative research and human studies of drug abuse. A rich history of animal research has implicated the NAcc as a critical substrate for craving and relapse in drug addiction.^40,41^ In rodents, virtually all drugs of abuse increase extracellular levels of dopamine in the NAcc, which putatively mediates their reinforcing effects.^13,42^ The current results support and extend these comparative findings by showing that NAcc activity is longitudinally associated with relapse to stimulant use in humans.

### Limitations

Several unresolved questions call for further investigation. Although the current study’s longitudinal design supports the inference of an association between neural responses to drug cues and relapse, the observed associations cannot establish causality. Neural markers indicating such an association might either precede or result from drug use. In the case of affective sensitization, neural markers indicating an association between responses to drug cues and relapse must have resulted from drug use, as association of drug cues with motivation requires at least 1 initial exposure.^43^ In the case of blunting, however, some evidence suggests that reduced responses to conventional reward cues may also predispose vulnerable individuals toward later substance abuse.^31^ Further research is needed to replicate and extend the findings to other samples (eg, women, as our sample of veterans included primarily men). Other similar designs will be necessary to assess whether the identified neural response is associated with other forms of addictive relapse. Although stimulant use clearly increases mesolimbic activity, this finding represents only one of many other types of addiction (eg, to nicotine, alcohol, or opiates). If people with opiate use disorder seek other types of hedonic experiences (eg, calm rather than excited positive affect), for instance, different neural responses to drug cues may be associated with relapse.^44^

## Conclusions

The current findings may highlight neural targets for intervention in stimulant use disorders. For instance, in rodent models, electrophysiological interference with NAcc activity can divert choices to consume highly palatable food.^45^ Future longitudinal studies might test whether temporally precise modifications of NAcc responses to drug cues can decrease the immediate or long-term risk of relapse in humans. Follow-up studies that integrate multimodal neural measures (eg, gray matter volume and white matter integrity) with more traditional measures (eg, self-report, behavioral, and clinical measures) may clarify when neuroimaging markers add value. Neural factors associated with relapse might also advance the development of new interventions. By implication, interventions that most effectively reduce NAcc activity (and associated appetitive motivation) in response to drug cues might diminish the risk of relapse. Finally, even in the absence of causal consequences, neural factors associated with relapse may help clinicians to direct interventions toward those at the greatest risk of relapse.

## Notes

Supplement. **eAppendix 1.** Supplementary Methods **eAppendix 2.** Supplementary Results **eFigure 1.** Participant Flow Diagram **eFigure 2.** Ratings of Cue Images in a Pilot Sample of Healthy Control Participants **eFigure 3.** Self-reported Responses to Different Cue Images in Patients vs Controls **eFigure 4.** Contrasts of Neural Responses to Food, Drug, and Neutral Trials in Patients and Controls **eFigure 5.** Receiver-Operating Characteristic (ROC) Curves of Models Predicting Relapse **eFigure 6.** Neural Features That Classify Relapse **eFigure 7.** Correlation of NAcc Activity With Self-reported Positive Arousal for Different Stimuli in Patients vs Controls **eTable 1.** Demographic Characteristics of Healthy Controls and Patients With a Stimulant Use Disorder **eTable 2.** Demographic and Clinical Characteristics of Early Relapsing vs Abstaining Patients **eTable 3.** Brain Regions With Significant Differences in Activation for Contrasts of Interest **eTable 4.** Logistic Regression Results of NAcc Response to Drug Cues Predicting Treatment Outcome at 1, 3, and 6 Months Posttreatment **eTable 5.** Whole-Brain Neural Features That Classify Relapse **eReferences**
