# Supplementary material for: Association of Neural Responses to Drug Cues With Subsequent Relapse to Stimulant Use
Source: JAMA Netw Open. 2018 Dec 28;1(8):e186466. doi: 10.1001/jamanetworkopen.2018.6466 (PMC6324538; doi:10.1001/jamanetworkopen.2018.6466)
Supplement: Supplement. — eAppendix 1. Supplementary Methods eAppendix 2. Supplementary Results eFigure 1. Participant Flow Diagram eFigure 2. Ratings of Cue Images in a Pilot Sample of Healthy Control Participants eFigure 3. Self-reported Responses to Different Cue Images in Patients vs Controls eFigure 4. Contrasts of Neural Responses to Food, Drug, and Neutral Trials in Patients and Controls eFigure 5. Receiver-Operating Characteristic (ROC) Curves of Models Predicting Relapse eFigure 6. Neural Features That Classify Relapse eFigure 7. Correlation of NAcc Activity With Self-reported Positive Arousal for Different Stimuli in Patients vs Controls eTable 1. Demographic Characteristics of Healthy Controls and Patients With a Stimulant Use Disorder eTable 2. Demographic and Clinical Characteristics of Early Relapsing vs Abstaining Patients eTable 3. Brain Regions With Significant Differences in Activation for Contrasts of Interest eTable 4. Logistic Regression Results of NAcc Response to Drug Cues Predicting Treatment Outcome at 1, 3, and 6 Months Posttreatment eTable 5. Whole-Brain Neural Features That Classify Relapse eReferences [file jamanetwopen-1-e186466-s001.pdf]

Supplementary Online Content

MacNiven KH, Jensen ELS, Borg N, Padula CB, Humphreys K, Knutson B. Association of neural responses to drug cues with subsequent relapse to stimulant use. *JAMA Netw Open.* 2018;1(8):e186466. doi:10.1001/jamanetworkopen.2018.6466

eAppendix 1. Supplementary Methods

eAppendix 2. Supplementary Results

eFigure 1. Participant Flow Diagram

eFigure 2. Ratings of Cue Images in a Pilot Sample of Healthy Control Participants

eFigure 3. Self-reported Responses to Different Cue Images in Patients vs Controls

eFigure 4. Contrasts of Neural Responses to Food, Drug, and Neutral Trials in Patients and Controls

eFigure 5. Receiver-Operating Characteristic (ROC) Curves of Models Predicting Relapse

eFigure 6. Neural Features That Classify Relapse

eFigure 7. Correlation of NAcc Activity With Self-reported Positive Arousal for Different Stimuli in Patients vs Controls

eTable 1. Demographic Characteristics of Healthy Controls and Patients With a Stimulant Use Disorder

eTable 2. Demographic and Clinical Characteristics of Early Relapsing vs Abstaining Patients

eTable 3. Brain Regions With Significant Differences in Activation for Contrasts of Interest

eTable 4. Logistic Regression Results of NAcc Response to Drug Cues Predicting Treatment Outcome at 1, 3, and 6 Months Posttreatment

eTable 5. Whole-Brain Neural Features That Classify Relapse

eReferences

This supplementary material has been provided by the authors to give readers additional information about their work.

© 2018 MacNiven KH et al. *JAMA Network Open.*

eAppendix 1. Supplementary Methods

Task images

Images were selected from files freely available on the internet and were pre-tested for affective impact. Drug images included pictures of crack, cocaine, methamphetamine and related paraphernalia (e.g., a crack pipe, cocaine on a mirror). Food images included pictures of appetizing food (e.g., cookies, pizza). Neutral images included pictures of common objects (e.g., stapler, computer mouse). All images depicted unbranded items and contained no text. To ensure that selected stimuli elicited predicted affective responses in healthy participants, affective ratings were collected in an internet survey on a separate sample of pilot control participants located in the United States (n=24). Findings from this sample confirmed that food cue images elicited higher ratings of wanting and positive arousal than did other cue images, as predicted (see eFigure 2).

Power analysis

Our main objective was to test the viability of using brain activity to predict relapse after treatment. We conducted a power analysis to determine an adequate patient sample size for addressing this question. Assuming a 50% base rate of relapse and an odds-ratio effect size of 2.63 (as previously reported in the context of alcohol relapse as a hazard ratio[1]), power calculation indicated that a one-tailed logistic regression test with alpha=.05 and power ($1-\beta$) = .80 would require 38 patients for a statistically valid sample. We aimed to enroll roughly the same number of controls as patients for group comparisons.

Handling the clinical risks associated with cue reactivity studies

Cue reactivity studies are designed to trigger craving responses for substances[2] and therefore can pose clinical risks to patients with substance use disorder. To mitigate against these risks,

the research team worked closely with the clinical treatment team to ensure any issues that arose during participation in the study were rapidly and adequately addressed. A member of the research team escorted patient participants between the treatment facility and study location to ensure safe transit. The treatment team was aware of the study protocol, including the cue-reactivity paradigm. Patients had access to social workers, case managers, psychologists and psychiatrists should they have needed supportive follow-up.

Follow-up assessments

Patients were notified that their participation would entail follow-up appointments one, three, and six months after the date of their treatment discharge. Follow-ups were not conducted with controls. At the end of the scanning session, patients completed a follow-up form requesting their contact information during this period, as well as information for two other close individuals (relatives, friends, or case worker) that could be contacted if the patient could not be reached. Patients then received three Amazon.com gift cards and were told that one card would be activated with $50 after completing each follow-up appointment.

Patients were contacted by phone, text, and/or email for follow-up appointment scheduling and reminders. If a patient could not be reached, then collateral sources (relatives, friends, and/or VA records) were contacted for information about how to reach the patient as well as their potential stimulant use. At each follow up, stimulant use was assessed using the Time-Line Follow-Back method [3]. This self-report measure of relapse shows moderate-to-high consistency with urine toxicology screens [4]. Patients were asked to identify any dates since their last appointment that they used stimulants. Due to the sensitive nature of self-reporting use of illicit substances, we limited our interview questions to date(s) of use and did not systematically record quantity of use for a given use day. The Brief Addiction Monitor questionnaire was also administered at each follow-up to assess recent (past month) use of other illicit substances.

© 2018 MacNiven KH et al. *JAMA Network Open.*

Follow-ups were conducted either via phone (80.8%) or in-person (19.2%), and urine toxicology screens were collected in a subset of the in-person interviews (53.3%). In every instance where urine drug samples were collected (n=8), they were consistent with patients' self-report[5]. Because treatment was abstinence-based, relapse was defined as any stimulant use in the time since treatment or the previous assessment. Relapse status was assigned based on patients' self-reported stimulant use in a follow-up interview or based on available medical records that explicitly noted a patient's use of stimulants (in no case did these sources conflict). Fourteen of the patients continued in some form of monitored program (either outpatient or residential) following treatment discharge, which provided an additional source of corroborating evidence of recovery outcomes.

Stimulant use data was successfully collected for all but one patient at the 1-month assessment. Two additional patients were lost to follow-up prior to the 3-month assessment, and 3 additional patients were lost prior to the final follow-up targeted at 6 months posttreatment discharge. Patients lost to follow-up (total n=6) did not significantly differ from other patients on any of the variables reported in eTable 2. In the interest of both minimizing the number of patients lost to follow-up and having a relatively equal number of relapsers and abstainers, we analyzed treatment outcome in the sample of 33 patients with either confirmed abstinence or confirmed relapse 3 months after treatment discharge. In this sample, the median follow-up duration was 194 days (SD=83.0; range, 90-463 days).

Neuroimaging scan acquisition

Scans were acquired with a 3 Tesla GE Discover MR750 scanner and a Nova Medical 32-channel head coil. Functional (T2*-weighted) images were acquired using an echo-planar imaging pulse sequence with the following parameters: TR=2 s, TE=25 ms, flip angle=77°,

© 2018 MacNiven KH et al. *JAMA Network Open.*

FOV=232 × 232, 80 × 80 acquisition matrix with 46 axial slices (no slice gap). Images were acquired in an interleaved order and voxel dimensions were 2.9 mm^3. Structural (T1-weighted) scans were acquired using GE's BRAVO sequence with the following parameters: TR=7.2 ms, TE=2.8 ms, flip angle=12°, FOV=256 × 230, 256 × 256 acquisition matrix with 186 slices, with slice thickness of 0.9 mm. Diffusion-weighted images were also acquired (described in a separate report).

Data processing and analysis

Pre-processing: the first six volumes of each functional scan were discarded to allow magnetization to reach steady-state. Images were then corrected for differences in slice acquisition times and head movement using Fourier interpolation, and smoothed using a 4 mm full-width at half maximum Gaussian kernel. Raw activity was then converted to percent signal change within each voxel and high-pass filtered to remove low frequency drift (admitting frequencies > 1 cycle per 90 s).

The first functional volume of each task was co-registered to a subject's T1-weighted anatomical volume in native space, and anatomical volumes were subsequently co-registered to an anatomical template (TT_N27) in standard Talairach space. These two transformations were combined and applied to functional data, aligning all participants' data in a common standardized space (i.e., Talairach space).

Regressors for whole-brain analyses: A general linear model was fit to each voxel time series that included task-related regressors (described below) as well as nuisance regressors. Nuisance regressors included six rigid-body movement parameters estimated during motion correction, as well as averaged activity time series extracted from white matter and cerebrospinal fluid VOIs [6]. To model the task, regressors were defined to model activity during

© 2018 MacNiven KH et al. *JAMA Network Open.*

the cue, image, and rating periods of each trial. Reaction time for ratings was also modeled as a regressor to capture signal variability associated with motor responses. Regressors of interest were then created which indicated each trial type (drug, food, alcohol, and neutral). For these regressors, each trial was modeled as an 8 second boxcar function over the entire trial duration, beginning with cue onset and terminating with the offset of the rating period. These regressors were then convolved using a single gamma function to account for hemodynamic lags (Cohen, 1996). Food vs neutral trials, drug vs neutral trials, and drug vs food trials were then contrasted, producing three contrast maps for each subject.

VOI definition: VOI masks of MPFC, NAcc, and VTA were created. The MPFC VOI was defined with an 8 mm diameter sphere bilaterally centered on Talairach coordinates ±5, 47, 0. The NAcc VOI was anatomically defined based on “Left-Accumbens” and “Right-Accumbens” labels in the Desai atlas. The VTA VOI was created based on a previously-described structural landmark demarcation of midbrain dopamine nuclei[7] (substantia nigra and VTA collectively). The bilateral VTA VOI was defined as the medial aspects of this mask, which included voxels spanning left and right coordinates from x=-5 to x=6. Because we had no predictions regarding laterality, we averaged left and right VOIs to reduce the number of statistical tests.

Whole-brain classification analysis: Binary classifiers were trained to distinguish relapsers from abstainers based on patients’ whole brain responses to drug cues. To maximize the number of instances for each class, we defined relapsers as the first half of participants in the patient sample to relapse, producing 15 relapsers and 15 abstainers at 215 days posttreatment discharge (only patients with at least six months of follow-up data were included in this analysis, leaving 30 patients). Features were selected using a support vector machine classifier with recursive feature elimination (SVM-RFE) in the python toolbox scikit-learn [8]. Features were defined as voxelwise regression beta coefficients modeling activity during drug cue trials.

© 2018 MacNiven KH et al. *JAMA Network Open.*

Beginning with all features within a whole brain gray matter mask, an SVM was trained to classify held-out test patients as a relapser or abstainer, using all other patients' data as the training set (LOSO cross-validation). Training sets were oversampled to contain even numbers of early abstainers and relapsers, setting the baseline probability of correctly classifying each held-out test subject to 50%. The least informative 5% of features (based on training set performance) were iteratively removed until <1% of features remained. This procedure was repeated for each regularization parameter C fixed at 0.001, .01, 0.1, 1.0, 10.0, 100.0, and 1000.0 to determine the best-performing combination of C value and percent features. After establishing the optimal combination of the c-parameter and feature cutoff, surviving features were projected back into brain space and documented (eFigure 6, eTable 5).

© 2018 MacNiven KH et al. *JAMA Network Open.*

eAppendix 2. Supplementary Results

Behavior

Mixed-level repeated measures analyses of variance (ANOVAs) confirmed that group (patient, control; between-subjects factor) and cue type (food, drug, neutral; within-subjects factor) influenced self-reported ratings collected both during and after the scan as predicted. Specifically, positive arousal ratings showed main effects of cue ($F(2,144)=123.7$, $p<.001$, $\varepsilon^2=.590$) and group ($F(1,144)=8.2$, $p=.006$, $\varepsilon^2=.006$), but not an interaction of cue X group ($F(2,144)=1.5$, $p=.221$, $\varepsilon^2=.007$). Similarly, negative arousal ratings showed main effects of cue ($F(2,144)=110.5$, $p<.001$, $\varepsilon^2=.592$) and group ($F(1,144)=4.1$, $p=.047$, $\varepsilon^2=.001$), but not an interaction of cue X group ($F(2,144)=.3$, $p=.770$, $\varepsilon^2=.001$). Want ratings, however, showed main effects of cue ($F(2,148)=130.1$, $p<.001$, $\varepsilon^2=.501$) and group ($F(1,148)=15.2$, $p<.001$, $\varepsilon^2=.027$), as well as an interaction of cue X group ($F(2,152)=13.6$, $p<.001$, $\varepsilon^2=.052$). Post hoc *t*-tests confirmed that all participants (i.e., both controls and patients) reported wanting food cues more than neutral cues (controls: $t(39)=5.77$, $p<.001$, $d= .91$; patients: $t(35)=6.55$, $p<.001$, $d=1.09$), and drug cues less than neutral cues (controls: $t(39)=-13.86$, $p<.001$, $d=2.19$; patients: $t(35)=-2.14$, $p< .001$, $d=-.36$). Patients, however, still reported wanting drug cues more than controls ($t(74)=5.61$, $p<.001$, $d=1.28$). Familiarity also showed main effects of cue ($F(2,144)=169.5$, $p<.001$, $\varepsilon^2=.320$) and group ($F(1,144)=57.9$, $p<.001$, $\varepsilon^2=.137$), as well as by an interaction of cue X group ($F(2,144)=125.6$, $p<.001$, $\varepsilon^2=.237$). Post hoc *t*-tests confirmed that patients reported more familiarity with drug cues than to controls ($t(72)=11.43$, $p<.001$, $d=2.63$). Similarly constructed cue X group ANOVA control analyses of average reaction times while entering ratings revealed no significant differences across groups or group by cue type interactions.

Relapse and NAcc drug responses by stimulant subgroups

As reported in Methods, our patient sample (n=36) consisted of 28 methamphetamine users and 14 crack or powder cocaine users (6 patients met the criteria for abusing more than one stimulant). These subgroups did not vary with respect to NAcc response to drug cues (two-sample *t* test: $t(40)=1.68$, $p=.10$, $d=.54$) nor with respect to relapse at 3 months (χ^2 test for equal proportions: $\chi^2(1, N=39) = 2.73$, $p=.10$).

© 2018 MacNiven KH et al. *JAMA Network Open.*

eFigure 1. Participant Flow Diagram

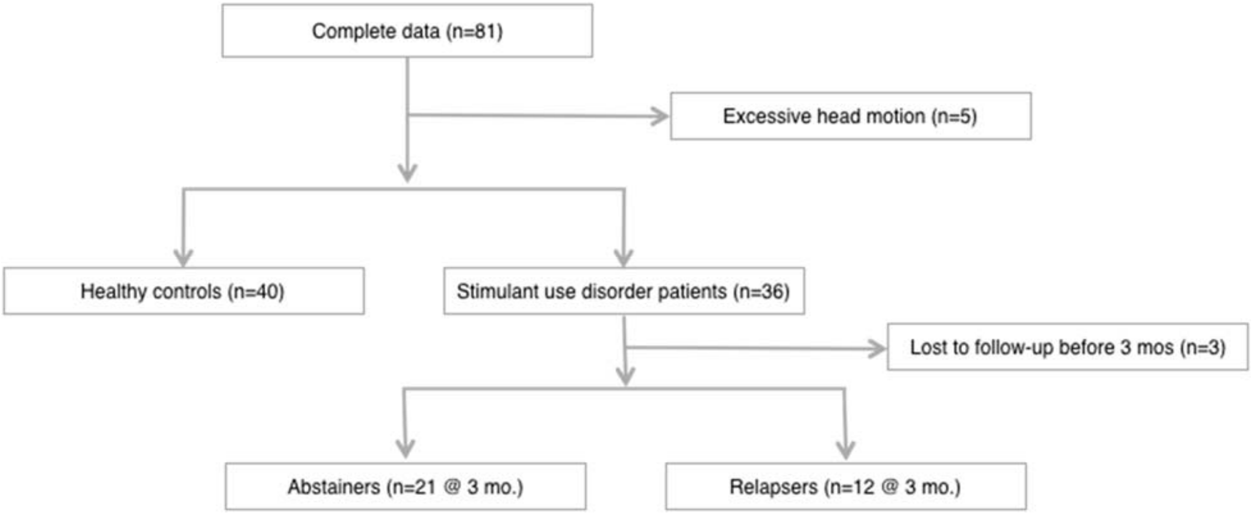

© 2018 MacNiven KH et al. *JAMA Network Open.*

eFigure 2. Ratings of Cue Images in a Pilot Sample of Healthy Control Participants

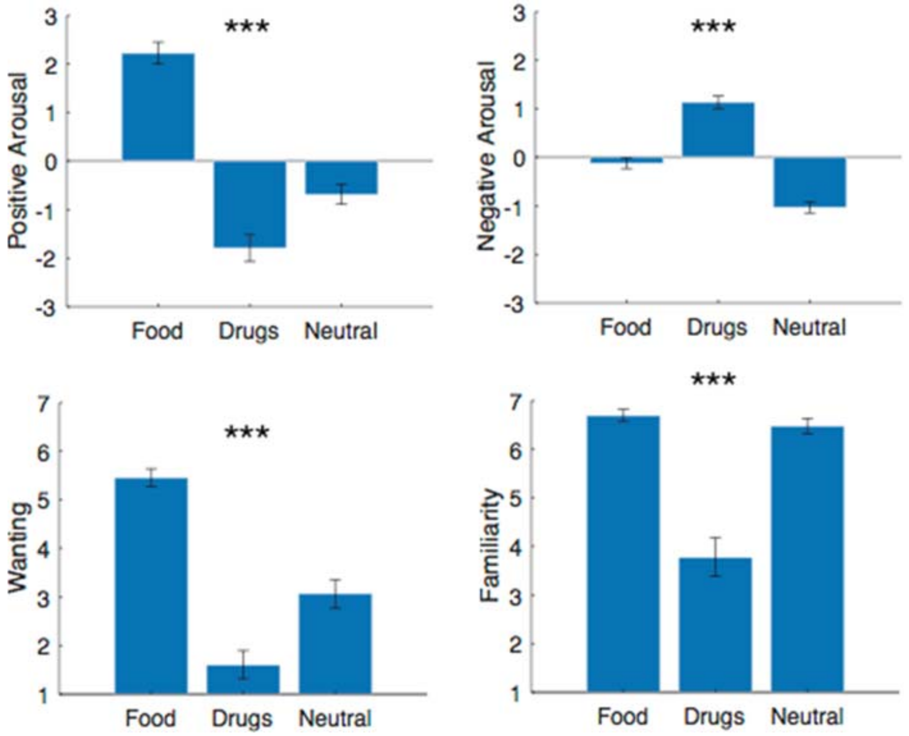

Affective ratings were collected from a separate group of participants (n=24) online using Amazon's Mechanical Turk platform. Participants were located in the United States and were between 18-60 years of age. Participants rated each image on 7-point scales indicating valence (from "very negative" to "very positive"), arousal (from "not at all aroused" to "highly aroused"), wanting (from "strongly don't want" to "strongly want"), and familiarity (from "not at all familiar" to "very familiar"). Valence and arousal ratings were then mean-deviated within subject and rotated to index positive arousal and negative arousal [9], consistent with the circumplex model of affect[10].

One-way ANOVAs revealed main effects of image type on all self-report measures. Positive arousal varied by image type $F(2,46)=56.1$; $p<.001$; $\varepsilon^2=.691$) and post-hoc *t*-tests indicated that food images were rated highest, followed by neutral images, with drug images rated lowest

© 2018 MacNiven KH et al. *JAMA Network Open.*

(food vs neutral: $t(23)=9.69$, $p<.001$, $d=1.98$; neutral vs drugs: $t(23)=2.76$, $p=.01$, $d=.56$). A similar pattern was observed for wanting ratings ($F(2,46)=54.9$; $p=.000$; $\varepsilon^2=.617$). Post-hoc *t*-tests confirmed that participants rated food images as more wanted than neutral images, and rated neutral images as more wanted than drug images (food vs neutral: $t(23)=7.36$, $p<.001$, $d=1.50$; neutral vs drugs: $t(23)=3.42$, $p=.002$, $d=.70$). Negative arousal ratings varied by image type ($F(2,46)=62.9$; $p<.001$, $\varepsilon^2=.723$) and post-hoc *t*-tests revealed that drug images were rated highest while neutral images were rated lowest on negative arousal, with food images falling in between (drugs vs food: $t(23)=6.31$, $p<.001$, $d=1.29$; food vs neutral: $t(23)=5.36$, $p<.001$, $d=1.09$). Familiarity also differed by image type ($F(2,46)=52.0$; $p<.001$; $\varepsilon^2=.54$). Post-hoc *t*-tests confirmed that food images were rated as most familiar, neutral images were rated as slightly less familiar, and drug images were rated as least familiar (food vs neutral: $t(23)=2.18$, $p=.04$, $d=.45$; neutral vs drugs: $t(23)=7.08$, $p<.001$, $d=1.45$).

© 2018 MacNiven KH et al. *JAMA Network Open.*

eFigure 3. Self-reported Responses to Different Cue Images in Patients vs Controls

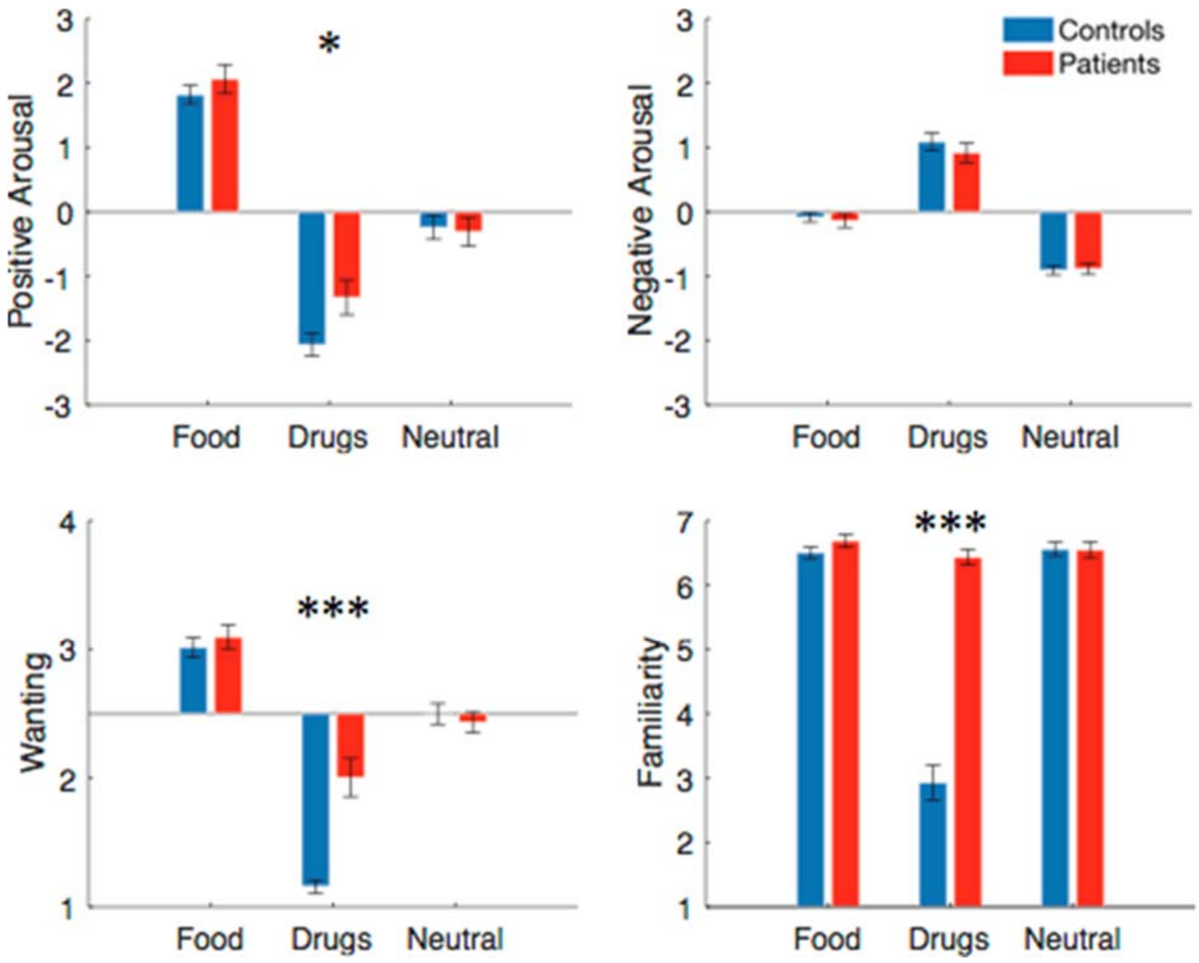

Average ratings of positive arousal (PA), negative arousal (NA), wanting, and familiarity for different image types (i.e., food, drug, neutral) for patients vs controls. Error bars depict ±s.e.m. across subjects. Group ratings differed at *p<.05, ***p<.001, two-tailed t-tests.

© 2018 MacNiven KH et al. *JAMA Network Open.*

eFigure 4. Contrasts of Neural Responses to Food, Drug, and Neutral Trials in Patients and Controls

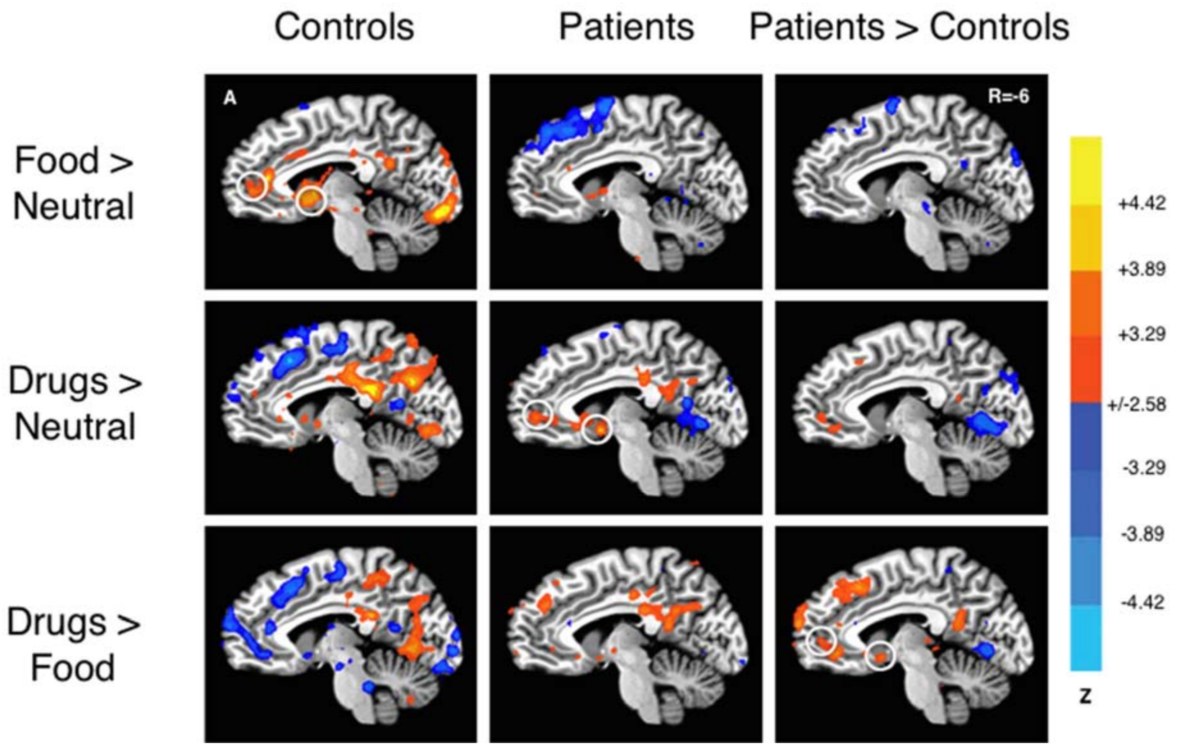

Whole-brain maps (sagittal view, 6 mm left of midline) show activations at a voxelwise threshold of $p<.01$ (uncorrected for display, each color increment represents an order of magnitude increase). Activation maps specifically depict contrasts for controls (left), patients (middle), and patients vs controls (right). Circles highlight predicted contrasts in MPFC and NAcc volumes of interest. A, anterior; R, right.

© 2018 MacNiven KH et al. *JAMA Network Open.*

eFigure 5. Receiver-Operating Characteristic (ROC) Curves of Models Predicting Relapse

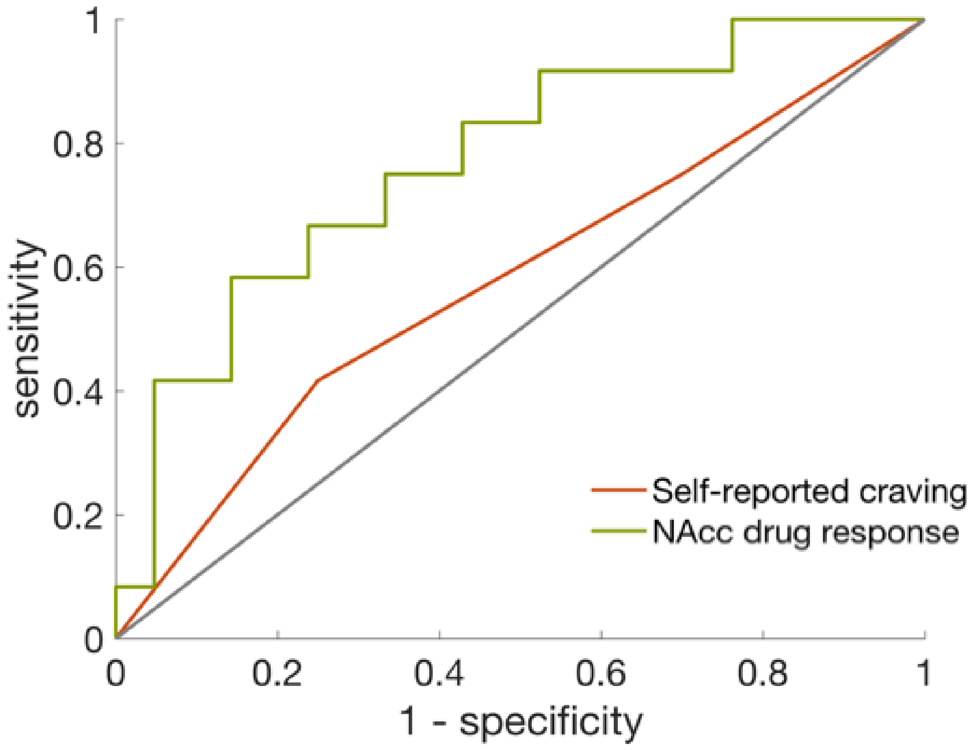

Curves illustrate the sensitivity and specificity of NAcc response to drug cues and self-reported craving in predicting relapse. Area under the curve (AUC) for NAcc drug response model is 77.0%; AUC for self-reported craving model is 57.7%.

eFigure 6. Neural Features That Classify Relapse

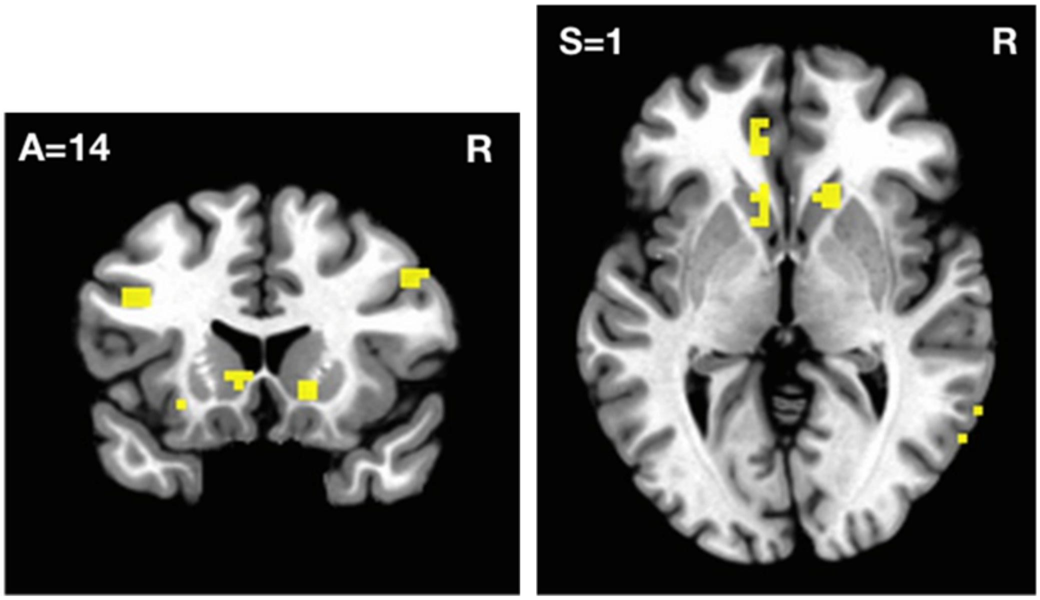

The highest classification accuracy of 60.0% (correctly classifying 18 out of 30 held-out test patients) was achieved with C=10.00 and used roughly 5% of features [11]. The figure shows these features back-projected into standardized brain space with a cluster threshold of at least 10 contiguous voxels. Features clustered within the ventral striatum (including NAcc) bilaterally (depicted in the left image in coronal view and the right image in an axial view). A full list of brain regions containing the top 5% most informative features are listed in eTable 5. A, anterior; R, right; S, superior.

© 2018 MacNiven KH et al. *JAMA Network Open.*

eFigure 7. Correlation of NAcc Activity With Self-reported Positive Arousal for Different Stimuli in Patients vs Controls

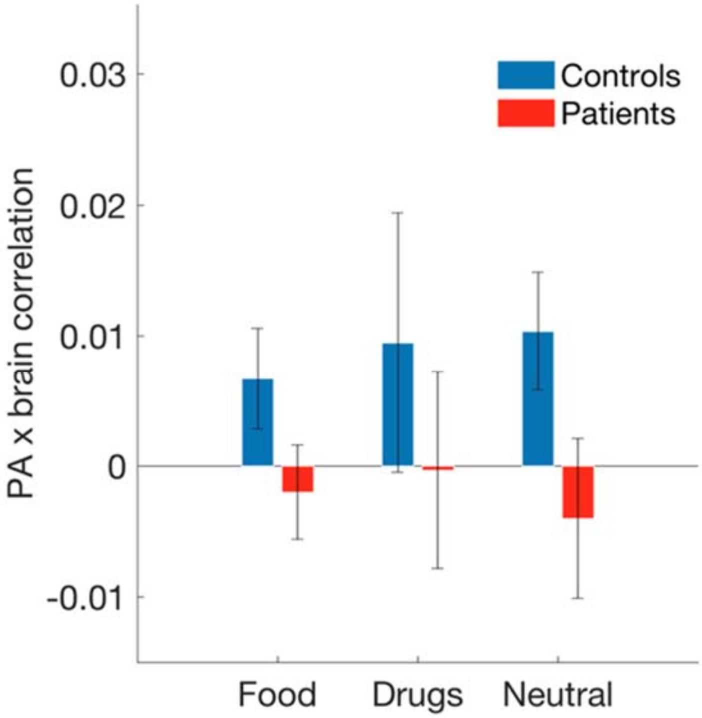

Parametric regressors weighted as a function of positive arousal ratings were created separately for food, drugs, and neutral stimuli. Seven controls and 6 patients had no variance in their ratings for drugs and so could not be included in this analysis. Mixed-level repeated measures analyses of variance (ANOVAs) indicated a main effect of group (patient, control; between-subjects factor) (F(1,122)=4.90, p=.03, ε^2=.023), but no significant effects of cue type (drug, food, neutral; within-subjects factor (F(2,122)=.10, p=.94), and no interaction of cue X group (F(2,122)=.10, p=.90). NAcc, nucleus accumbens; PA, positive arousal.

© 2018 MacNiven KH et al. *JAMA Network Open.*

eTable 1. Demographic Characteristics of Healthy Controls and Patients With a Stimulant Use Disorder

		**Controls (n=40)**	**Patients (n=36)**
Age (mean±sd years)*		32.0±11.6	43.4±13.3
Sex (% male)*		60%	94%
Race			
Asian		28%	14%
African-American		3%	17%
Caucasian		53%	40%
Hispanic		10%	20%
Education (mean±sd years completed)*		15.9±2.8	12.9±1.5
% Smokers*		12%	69%
% Veterans*		28%	100%
BDI scores (mean±sd)*		6.6±6.7	16.0±11.6
BIS scores (mean±sd)*		58.7±8.5	72.6±12.2
Discount rate (mean±sd of log(k))*		-4.87±1.56	-3.80±1.41

Between group comparisons were by *t*-test.
* *p*<.05, uncorrected
BDI, Beck Depression Inventory; BIS, Barratt Impulsiveness Scale.

© 2018 MacNiven KH et al. *JAMA Network Open.*

eTable 2. Demographic and Clinical Characteristics of Early Relapsing vs Abstaining Patients

		Early relapsers (n=12)	Early abstainers (n=21)
Age (mean±sd years)*		49.3±14.1	39.3±12.3
Sex (% male)		92%	95%
Race			
Asian		17%	15%
African-American		33%	10%
Caucasian		33%	40%
Hispanic		8%	25%
Education (mean±sd years completed)		13.3/2.0	12.6/1.1
% Smokers		50%	81%
BDI scores (mean±sd)		18.7±12.2	15.2±11.9
BIS scores (mean±sd)		73.8±11.3	72.0±11.4
Discount rate (mean±sd of log(k))		-3.69±1.49	-3.73±1.19
% PTSD diagnosis		33%	52%
% Anxiety diagnosis		25%	10%
% Depression diagnosis		25%	38%
% Alcohol dependence [a]		50%	52%
% Marijuana use [b]		67%	52%
% Opiate dependence [c]		8%	24%
% Poly-drug dependence (including alcohol, not including nicotine)		83%	81%
Years of stimulant use (mean±sd)		21.5±13.1	15.3±12.5
Days sober prior to participation (median±sd)		28.0±80.9	29.0±58.3

© 2018 MacNiven KH et al. *JAMA Network Open.*

Days in rehab prior to participation (mean±sd)		15.7±7.9	20.0±8.9

Between group comparisons were by *t*-test.
* *p*<.05, uncorrected
BDI, Beck Depression Inventory; BIS, Barratt Impulsiveness Scale; PTSD, Post-traumatic stress disorder.
[a] defined as score of 4 or higher on Alcohol Use Disorders Identification Test alcohol consumption questions (AUDIT-C)[12]
[b] based on recent use (i.e., month prior to treatment)
[c] diagnosis based on DSM-5 criteria

eTable 3. Brain Regions With Significant Differences in Activation for Contrasts of Interest

Region	x	y	z	Peak Z	Voxels
**Food > Neutral**					
Patients					
Left & Right NAcc[a]	±10	13	-2	2.757	20
Left Inferior Temporal Gyrus	-42	-72	0	-5.356	653
Right Fusiform Gyrus	42	-46	-18	-5.118	325
Left Inferior Frontal Gyrus	-48	30	-3	-4.911	297
Left Middle Frontal Gyrus	-28	1	52	-5.266	249
Right Lingual Gyrus	25	-69	-15	5.249	147
Left Medial Frontal Gyrus	-4	27	46	-4.667	106
Left Lingual Gyrus	-16	-89	0	5.506	64
Left Fusiform Gyrus	-36	-34	-18	-5.019	61
Left Superior Frontal Gyrus	-7	-2	67	-4.675	33
Controls					
Left & Right MPFC[a]	±4	46	1	3.670	24
Left & Right NAcc[a]	±10	13	-2	5.035	20
Left & Right VTA[a]	±3	-16	-11	3.081	26
Left Lingual Gyrus	-16	-86	-6	7.307	590
Right Lingual Gyrus	25	-69	-9	7.382	565
Right Insula	36	7	-6	6.024	320
Left Middle Temporal Gyrus	-45	-63	17	-4.889	222
Left Anterior Cingulate	-4	36	14	5.218	214
Left Insula	-36	4	-6	5.810	155
Right Middle Temporal Gyrus	45	-66	6	-5.325	153
Right Superior Frontal Gyrus	19	65	14	4.437	74

© 2018 MacNiven KH et al. *JAMA Network Open.*

Right Cingulate Gyrus	4	-28	38	4.610	71
Left Middle Frontal Gyrus	-22	36	-6	5.644	69
Right Posterior Cingulate	4	-46	23	4.384	69
Left Nucleus Accumbens	-10	10	-3	4.776	67
Right Inferior Parietal Lobule	51	-51	40	4.130	57
Left Middle Frontal Gyrus	-39	33	-3	-4.880	56
Right Middle Frontal Gyrus	36	36	14	5.513	56
Right Middle Frontal Gyrus	22	30	-12	4.451	47
Right Parahippocampal Gyrus	25	-46	-6	4.070	26
Patients > Controls					
Right Superior Frontal Gyrus	10	27	52	-4.590	61
**Drugs > Neutral**					
Patients					
Left & Right MPFC[a]	±4	46	1	3.075	24
Left & Right NAcc[a]	±10	13	-2	3.263	20
Right Middle Occipital Gyrus	30	-74	8	5.282	149
Left Middle Occipital Gyrus	-33	-77	12	5.193	140
Right Parahippocampal Gyrus	28	-54	-6	5.750	135
Left Fusiform Gyrus	-28	-54	-9	4.716	61
Right Cingulate Gyrus	1	-22	35	4.649	57
Left Middle Temporal Gyrus	-45	-34	-3	-4.399	49
Left Middle Occipital Gyrus	-39	-60	-3	4.029	31
Right Fusiform Gyrus	42	-34	-15	4.547	28
Left Posterior Cingulate	-7	-57	14	-4.515	27
Right Insula	39	-2	0	4.968	26
Controls					

© 2018 MacNiven KH et al. *JAMA Network Open.*

Left Middle Occipital Gyrus	-28	-77	12	6.637	3727
Right Fusiform Gyrus	22	-69	-9	7.022	459
Left Middle Frontal Gyrus	-42	36	-3	-6.621	338
Left Superior Frontal Gyrus	-16	24	61	-6.100	262
Left Inferior Parietal Lobule	-57	-31	38	5.645	226
Right Postcentral Gyrus	28	-28	64	-4.678	217
Left Precentral Gyrus	-28	-28	61	-5.370	217
Right Middle Frontal Gyrus	28	47	20	5.433	131
Right Cerebellar Tonsil	36	-63	-38	-4.968	70
Right Anterior Cingulate	7	36	17	4.144	35
Left Medial Frontal Gyrus	-2	-11	52	-4.121	34
Right Middle Frontal Gyrus	42	4	55	4.071	32
Right Middle Frontal Gyrus	25	27	38	4.371	31
Right Inferior Frontal Gyrus	33	21	-15	5.097	28
Right Insula	39	15	12	4.573	28
Right Cingulate Gyrus	1	15	26	3.943	26
Right Lingual Gyrus	16	-86	3	5.171	25
Right Medial Frontal Gyrus	4	42	32	4.585	24
Patients > Controls					
Right Supramarginal Gyrus	59	-46	32	-5.297	159
Left Lingual Gyrus	-10	-69	-3	-5.359	108
Right Culmen	13	-60	-6	-4.287	72
Right Middle Temporal Gyrus	62	-34	0	-3.993	32
Right Superior Frontal Gyrus	25	47	17	-3.992	25
**Drugs > Food**					
Patients					

© 2018 MacNiven KH et al. *JAMA Network Open.*

Right Middle Occipital Gyrus	45	-63	-6	5.895	1268
Left Middle Occipital Gyrus	-39	-72	8	6.244	1007
Left Cingulate Gyrus	-2	-8	35	4.450	102
Left Precuneus	-25	-48	40	4.485	77
Right Middle Frontal Gyrus	45	10	32	4.284	76
Left Inferior Parietal Lobule	-60	-31	38	4.836	50
Right Cuneus	19	-92	8	-4.904	46
Left Lingual Gyrus	-16	-92	-0	-4.390	41
Right Middle Frontal Gyrus	42	27	17	4.097	36
Right Middle Frontal Gyrus	30	-8	46	4.028	25
Controls					
Left & Right MPFC[a]	±4	46	1	-2.493	24
Left & Right NAcc[a]	±10	13	-2	-2.753	20
Left & Right VTA[a]	±3	-16	-11	-3.651	26
Left Middle Temporal Gyrus	-39	-63	6	7.285	1690
Right Middle Occipital Gyrus	45	-66	-3	6.823	1238
Right Cuneus	16	-92	6	-6.734	348
Left Lingual Gyrus	-16	-89	-3	-6.769	312
Right Fusiform Gyrus	39	-46	-12	5.454	279
Left Inferior Parietal Lobule	-60	-31	29	6.431	168
Right Precuneus	4	-51	40	4.684	164
Left Middle Frontal Gyrus	-33	36	-6	-4.979	143
Left Medial Frontal Gyrus	-10	15	46	-4.533	115
Left Superior Frontal Gyrus	-10	62	23	-4.588	69
Right Cerebellar Tonsil	36	-60	-35	-4.810	59
Left Postcentral Gyrus	-36	-25	46	-4.030	58

© 2018 MacNiven KH et al. *JAMA Network Open.*

Left Inferior Parietal Lobule	-28	-43	40	4.346	43
Patients > Controls					
Left & Right NAcc[a]	±10	13	-2	2.528	20
Left & Right VTA[a]	±3	-16	-11	3.127	26
Left Middle Frontal Gyrus	-33	33	-6	4.601	70
Left Medial Frontal Gyrus	-2	24	43	4.347	30
Left Lingual Gyrus	-7	-69	-3	-4.632	26

For whole brain map results, voxels were thresholded at p<.001 (uncorrected) and cluster-level thresholded at 23 or more contiguous voxels to produce clusters significant at p<.05, corrected. For volumes of interest, statistics were Bonferroni corrected for tests in three bilateral mesolimbic regions, such that only results at p <.0167 are reported. Coordinates are in Talairach space.

MPFC, medial prefrontal cortex; NAcc, nucleus accumbens; VTA, ventral tegmental area.
[a]VOI-based analysis; voxels within VOI mask were averaged to produce a single *Z*-score for each VOI. Reported coordinates are the mean of each VOI mask.

© 2018 MacNiven KH et al. *JAMA Network Open.*

eTable 4. Logistic Regression Results of NAcc Response to Drug Cues Predicting Treatment Outcome at 1, 3, and 6 Months Posttreatment[a]

	**1 month**	**3 months**	**6 months**
n relapsers / *n* patient sample[b]	9 / 35	12 / 33	15 / 30
Intercept	-1.25±0.45**; -2.78	-0.69±0.42; -1.61	0.08±0.45; 0.19
NAcc drug response	0.95±0.43*; 2.19	1.24±0.48**; 2.57	1.52±0.60*; 2.51
Pseudo R^2	0.162	0.246	0.277
AIC	38.45	38.49	35.31
Classification Accuracy (LOSO %)[c]	71.4%*	72.7%**	66.7%*

Statistics are standardized regression coefficients ± standard errors; followed by *Z*-scores.
*p<.05; ** p<.01
NAcc, nucleus accumbens; AIC, Akaike information criterion.
[a] Patients who relapsed within 40, 100, and 220 days posttreatment were considered relapsers by 1, 3, and 6 months posttreatment, respectively.
[b] Only patients with confirmed abstinence or relapse at the target date were included each analysis (i.e., patients lost to follow-up were excluded for relevant time periods).
[c] Classification accuracy was calculated based on leave-one-subject-out (LOSO) cross-validation. Training sets were oversampled to contain even numbers of abstainers and relapsers, setting the baseline probability of correctly classifying each held-out test patient to 50%.

© 2018 MacNiven KH et al. *JAMA Network Open.*

eTable 5. Whole-Brain Neural Features That Classify Relapse

**Region**	**x**	**y**	**z**	**Peak feature weight, *w***	**Voxels**
Left Cingulate Gyrus	-10	-54	29	.002	96
Right Middle Temporal Gyrus	42	-60	26	.002	78
Left Middle Frontal Gyrus	-48	30	29	.002	58
Left Uncus	-30	-16	-29	.002	48
Left Precuneus	-19	-57	32	.002	41
Left Cingulate Gyrus	-2	-25	38	.002	37
Right Uncus	36	-8	-29	.002	30
Right Superior Frontal Gyrus	19	50	-9	.002	27
Right Medial Frontal Gyrus	16	-11	55	-.002	27
Left Middle Temporal Gyrus	-42	-74	29	.002	26
Left Cerebellum	-19	-74	-18	-.002	25
Left Inferior Parietal Lobule	-45	-54	43	.002	25
Right Middle Frontal Gyrus	48	30	26	.002	24
Left Superior Frontal Gyrus	-25	50	26	.001	23
Right Inferior Temporal Gyrus	59	-54	-6	.002	20
Right Cingulate Gyrus	7	30	26	.002	19
Left Inferior Parietal Lobule	-54	-37	43	.002	19
Left Nucleus Accumbens	-7	10	-3	.002	18
Right Precuneus	7	-63	35	.001	16
Left Insula	-30	12	-6	.002	15
Right Nucleus Accumbens	16	18	-3	.002	15
Left Insula	-45	10	14	.001	15
Right Cingulate Gyrus	7	-40	35	.002	15
Right Precuneus	28	-69	49	.002	13
Left Anterior Cingulate	-10	39	0	.002	12

© 2018 MacNiven KH et al. *JAMA Network Open.*

Right Superior Temporal Gyrus	48	-46	14	.002	12
Left Middle Temporal Gyrus	-48	-66	20	-.002	12
Right Superior Frontal Gyrus	13	56	35	-.001	12
Left Middle Temporal Gyrus	-45	-74	14	.002	11
Left Superior Frontal Gyrus	-36	42	32	.002	11
Right Cerebellum	16	-69	-18	-.002	10
Right Inferior Parietal Lobule	65	-31	26	-.002	10

The 5% most informative features were selected using an SVM-RFE classifier with C parameter of 10.00 and then back-projected into standardized brain space (Talairach warped) and cluster-thresholded at 10 voxels. Positive weights indicate greater drug cue-induced activity for relapsers, while negative weights indicate greater drug cue-induced activity for abstainers. Coordinates are in Talairach space.

© 2018 MacNiven KH et al. *JAMA Network Open.*

eReferences

1. Reinhard, I. *et al.* A comparison of region-of-interest measures for extracting whole brain data using survival analysis in alcoholism as an example. *J. Neurosci. Methods* **242,** 58–64 (2015).
2. Drummond, D. C. What does cue-reactivity have to offer clinical research? *Addiction* **95,** 129–144 (2000).
3. Sobell, L. C. & Sobell, M. B. Timeline Follow-Back. in *Measuring Alcohol Consumption* 41–72 (1992). doi:10.1007/978-1-4612-0357-5_3
4. Hersh, D., Mulgrew, C. L., Van Kirk, J. & Kranzler, H. R. The validity of self-reported cocaine use in two groups of cocaine abusers. *J. Consult. Clin. Psychol.* **67,** 37–42 (1999).
5. Calhoun, P. S. *et al.* Drug use and validity of substance use self-reports in veterans seeking help for posttraumatic stress disorder. *J. Consult. Clin. Psychol.* **68,** 923–927 (2000).
6. Chang, C. & Glover, G. H. Effects of model-based physiological noise correction on default mode network anti-correlations and correlations. *Neuroimage* **47,** 1448–59 (2009).
7. Hennigan, K., D'Ardenne, K. & McClure, S. M. Distinct Midbrain and Habenula Pathways Are Involved in Processing Aversive Events in Humans. *J. Neurosci.* **35,** 198–208 (2015).
8. Pedregosa, F. *et al.* Scikit-learn: Machine Learning in Python. *J. Mach. Learn. Res.* **12,** 2825–2830 (2012).
9. Knutson, B., Katovich, K. & Suri, G. Inferring affect from fMRI data. *Trends Cogn. Sci.* **18,** 422–8 (2014).
10. Watson, D., Wiese, D., Vaidya, J. & Tellegen, A. The two general activation systems of affect: Structural findings, evolutionary considerations, and psychobiological evidence. *J. Pers. Soc. Psychol.* **76,** 820–838 (1999).
11. Ferenczi, E. A. *et al.* Prefrontal cortical regulation of brainwide circuit dynamics and

© 2018 MacNiven KH et al. *JAMA Network Open.*

reward-related behavior. *Science (80-.).* **351,** aac9698-aac9698 (2016).

12. Bush, K. *et al.* The audit alcohol consumption questions (audit-c): An effective brief screening test for problem drinking. *Arch. Intern. Med.* **158,** 1789–1795 (1998).
